# Dorsal Anterior Cingulate Cortex Responses to Repeated Social Evaluative Feedback in Young Women with and without a History of Depression

**DOI:** 10.3389/fnbeh.2016.00064

**Published:** 2016-03-31

**Authors:** Katarina Dedovic, George M. Slavich, Keely A. Muscatell, Michael R. Irwin, Naomi I. Eisenberger

**Affiliations:** ^1^Social and Affective Neuroscience Laboratory, University of CaliforniaLos Angeles, Los Angeles, CA, USA; ^2^Addiction Research Studies and Laboratory, Douglas Hospital Research Centre, McGill UniversityMontreal, QC, Canada; ^3^Cousins Center for Psychoneuroimmunology and Department of Psychiatry and Biobehavioral Sciences, University of CaliforniaLos Angeles, Los Angeles, CA, USA; ^4^The Robert Wood Johnson Foundation Health and Society Scholars Program, University of California, San Francisco/University of CaliforniaBerkeley, San Francisco, CA, USA; ^5^Department of Psychology, University of CaliforniaBerkeley, Berkeley, CA, USA

**Keywords:** social evaluation, social rejection, dorsal anterior cingulate cortex, major depressive disorder, vulnerability, resilience

## Abstract

The dorsal anterior cingulate cortex (dACC) is recruited when a person is socially rejected or negatively evaluated. However, it remains to be fully understood how this region responds to repeated exposure to personally-relevant social evaluation, in both healthy populations and those vulnerable to Major Depressive Disorder (MDD), as well as how responding in these regions is associated with subsequent clinical functioning. To address this gap in the literature, we recruited 17 young women with past history of MDD (previously depressed) and 31 healthy controls and exposed them to a social evaluative session in a neuroimaging environment. In two bouts, participants received an equal amount of positive, negative, and neutral feedback from a confederate. All participants reported increases in feelings of social evaluation in response to the evaluative task. However, compared to healthy controls, previously depressed participants tended to show greater increases in depressed mood following the task. At the neural level, in response to negative (vs. positive) feedback, no main effect of group or evaluation periods was observed. However, a significant interaction between group and evaluation periods was found. Specifically, over the two bouts of evaluation, activity in the dACC decreased among healthy participants while it increased among previously depressed individuals. Interestingly and unexpectedly, in the previously depressed group specifically, this increased activity in dACC over time was associated with lower levels of depressive symptoms at baseline and at 6-months following the evaluation session (controlling for baseline levels). Thus, the subset of previously depressed participants who showed increases in the recruitment of the dACC over time in response to the negative evaluation seemed to fair better emotionally. These findings suggest that examining how the dACC responds *to repeated bouts* of negative evaluation reveals a new dimension to the role of the dACC in processing exclusion and contributing to mental health outcomes in a population vulnerable to MDD. Further, investigation of the dynamics of the dACC response to negative social evaluation is warranted.

## Introduction

Major Depressive Disorder (MDD) is a severe, debilitating disorder, affecting approximately twice as many women compared to men (Marcus et al., 2012; Ferrari et al., 2013). It is characterized by the presence of depressed mood and/or loss of interest for at least 2 weeks, along with a combination of several psychophysiological symptoms such as sleep disturbances, fatigue, poor concentration, and feelings of guilt/worthlessness, which all contribute to impaired social and occupational functioning (American Psychiatric Association, 2000).

Experiences of psychological stress, particularly social stressors such as social evaluation or social rejection, are intricately linked with the development of depression. Specifically, individuals experiencing social rejection are 22 times more likely to develop depression (Kendler et al., 2003), and do so more quickly (Slavich et al., 2009), than persons not experiencing such stress. It has been suggested that maladaptive responses to social rejection at the neural, psychological, and physiological levels interact with each other as well as with other vulnerability factors, such as past history of depression, levels of early life stress, and genetic factors, to increase a person's vulnerability to depression (Slavich et al., 2010). Notably, being able to adaptively respond to *repeated* experiences of psychological stress is an important aspect of one's vulnerability or resilience to MDD (Southwick et al., 2005). However, the neural mechanisms subserving this dynamic process remain unclear. Here, we addressed this question by exposing female participants with and without prior history of MDD to two bouts of social evaluation in a neuroimaging environment.

Past history of MDD is an important moderator of the association between experiences of social evaluation and vulnerability to depression. Indeed, while the onset of the first lifetime depressive episode is tightly linked with highly stressful life experiences, it has been shown that once the first depressive episode has been experienced, subsequent episodes can be triggered by much milder stressors (Stroud et al., 2011), especially interpersonal stressors (Slavich et al., 2011). Moreover, with each new depressive episode experienced, the risk for subsequent episodes increases (Burcusa and Iacono, 2007; Koppers et al., 2011). Therefore, prior history of depression is an important context in which to examine mechanisms underlying the link between repeated experiences of social evaluation and rejection and subsequent risk for depression.

Previous studies in healthy samples have shown that when a person experiences social rejection or negative evaluation compared to social acceptance or positive evaluation, there is heightened activity in the dorsal anterior cingulate cortex (dACC; Eisenberger et al., 2003, 2011; Kross et al., 2011; Rotge et al., 2015); c.f., (Somerville et al., 2006). The dACC has been proposed to be part of the “neural alarm system” (Eisenberger and Lieberman, 2004; Spunt et al., 2012) and as such is involved in both detection and appraisal of social exclusion (Kawamoto et al., 2015), which are dynamic processes.

Along these lines, it has recently been suggested that dACC activity changes over the course of an episode of social rejection or exclusion (Kawamoto et al., 2015; Rotge et al., 2015). Specifically, several studies focusing primarily on event-related potentials have reported that, in healthy individuals, activity in dACC decreases over repeated exposure to social rejection (Crowley et al., 2009; Moor et al., 2012; Kawamoto et al., 2013; Themanson et al., 2013). For example, it was observed that in healthy young adults, across two sets of 20 exclusion trials each, N2 (reflecting ACC—based neural alarm activation) and P3b components (reflecting conscious cognitive control and attentional processes), were larger during the first 20 complete-exclusion event trials compared to the second 20 in a computerized game of social exclusion called Cyberball (Themanson et al., 2013). Similarly, Kawomoto and colleagues investigated the P3b component in healthy adults and observed a decrease in amplitude in the second half compared to first half of exclusion period of Cyberball (Kawamoto et al., 2013). Furthermore, an fMRI study with healthy adolescents and young adults observed that dACC activity was higher during the first block of exclusion compared to the middle or last block of exclusion in Cyberball (Moor et al., 2012). Overall, these findings suggest that, in healthy individuals, dACC activity decreases over periods of negative social experiences.

With depressed individuals, however, the overall response of the dACC is more mixed. Specifically, a meta-analysis found a hyperactive dACC response to processing negative information in MDD individuals (Hamilton et al., 2011; Graham et al., 2013), while other studies using more cognitive tasks showed decreased dACC activity in MDD (e.g., Crocker et al., 2013; Ubl et al., 2015). Another meta-analysis revealed that patients with MDD compared to controls show overall heightened levels of dACC activity across many study paradigms (Graham et al., 2013). With respect to the temporal dynamics of the dACC response, one study investigated activity in medial prefrontal cortex including dACC in response to a social evaluative threat task in depressed individuals with and without co-morbid anxiety compared to controls and individuals with anxiety (Waugh et al., 2012). In this study, participants were first asked to relax for 2 min, then to prepare to give a speech for another 2 min, and finally to simply relax since in the end they would not need to give a speech. The authors observed that while all depressed individuals exhibited a resurgence of medial frontal cortex including dACC activation during the late speech preparation period, participants without depression (controls and those with non-comorbid anxiety) exhibited a return to baseline during this period (Waugh et al., 2012). Thus, depressed individuals may show an increase in dACC activity over the course of a stressful task.

In healthy individuals, overall increased activity in dACC tracks with key psychological factors associated with vulnerability to depression, such as interpersonal sensitivity and low self-esteem such that the higher the levels of the vulnerability factor, the greater the overall dACC activity in response to rejection over acceptance (e.g., Eisenberger et al., 2003, 2011; Kong et al., 2015; Rotge et al., 2015). With respect to dynamic change in dACC activity, one study revealed that it is also associated with psychological variables. Specifically, Themanson and colleagues have observed that the increase in P3b amplitude from inclusion to the initial exclusion phase of Cyberball was associated with less positive affect and less feelings of control (Themanson et al., 2013). In MDD, the associations between the dynamic dACC response and psychological measures have not been explored (Waugh et al., 2012).

While all these studies offer insight into the link between vulnerability to MDD and nature of dACC activity in response to processing various types of negative information, there is an absence of empirical research examining whether past experience of depression is associated with a differential activity in the dACC particularly in response to repeated personally-relevant social evaluation, an important aspect of vulnerability to MDD. In addition, it remains unclear how, in this population, does the change of activity over repeated bouts of social evaluation in dACC track with psychological responses to social evaluation, as well as current and future clinical functioning?

To address some of the gaps in the current literature with respect to the association between repeated experiences of social evaluation and subsequent vulnerability to depression, the current study exposed young women with a past history of depression (previously depressed) and healthy controls to two bouts of social evaluation in a Magnetic Resonance Imaging (MRI) scanner. We focused on female participants due to the fact that: (a) approximately twice as many women compared to men suffer from depression (Marcus et al., 2012; Ferrari et al., 2013) and (b) women are particularly sensitive to interpersonal stressors (Stroud et al., 2002). We expected that, compared to the controls, previously depressed participants would show overall greater activity in dACC in response to negative (vs. positive) social-evaluative feedback. In addition, we hypothesized that whereas previously depressed participants would show an increase in activity in the dACC over the two exposures to social evaluation, controls would show a decrease. Furthermore, we also explored how changes in activity in the dACC in response to repeated bouts of social evaluation related to psychological responses to social evaluation, current clinical functioning, as well as vulnerability to depression at 6 and 12 months following the evaluation. We expected that the increase in dACC activity over the course of the social evaluative session in previously depressed sample would be related to poorer psychological and clinical outcomes.

## Methods and materials

### Subject selection

General eligibility criteria for participation in this study were: (a) being female; (b) aged between 18 and 25 years; (c) being right handed; (d) meeting safety criteria to participate in functional MRI (fMRI) research; (e) not having present or past history of autoimmune, liver or other severe chronic diseases; (f) not using hormonal contraception; and (g) no substance/alcohol abuse in the past 6 months. Additionally, participants in the control group needed to meet the following eligibility criteria: (a) no current or past history of any Axis I disorder, including MDD, and (b) no current or past history of taking psychiatric medication. Specific eligibility criteria for the previously depressed group were: (a) having had one or two lifetime major depressive episodes; (b) no Major Depressive Episode or major Axis I disorder in the past month; (c) no history of chronic, unremitting depression; (d) no psychiatric medication in past month; and (e) no past history of Post-traumatic Stress Disorder, mania, psychosis, delusions, or bipolar disorder. Consistent with other related studies (e.g., LeMoult et al., 2009; Harkness et al., 2010), potential previously depressed participants were not excluded if they had a history of general anxiety disorder, social anxiety disorder, or dysthymia in addition to depression.

### Procedure

Through university online classifieds and campus posters, we recruited 17 previously depressed and 31 control females to participate in a study that examined “the link between how the brain and body respond to first impressions and vulnerability to depression.” Initial eligibility was established during a telephone-screening interview and was verified on a separate day, in an in-person session, via a Structured Clinical Interview for DSM Diagnosis (SCID; First et al., 1995). During this time, participants also completed a screener for safety criteria for participation in fMRI research. Eligible participants then completed a personally relevant interview—the “impressions interview”—that was videotaped (to be used later for the social evaluative session). Within 1–3 days following the impressions interview, subjects completed the fMRI safety screening again and then participated in an fMRI scanning session. The fMRI testing session always occurred within 2 weeks of the SCID. Furthermore, participants completed follow-up questionnaires online at ~6 and 12 months following the fMRI session to assess their depressive symptoms during the year following the baseline study visits. The Institutional Review Board of the University of California, Los Angeles approved the study, and all participants provided written informed consent.

### Impressions interview

Participants were told that, in order to examine how the brain and body respond to first impressions, all participants first needed to complete an “impressions interview.” The interview consisted of answering personally relevant questions while being videotaped, for approximately 10 min. Some of the questions included “What are you most proud of that you have done in your life so far?” or “What are some of your shortcomings?.” They were also informed that, on the scan day, they would be paired with another participant, and at that time, the experimenters would choose one of them to form an impression of the other based on the video of the interview. Meanwhile, the other person would be scanned while they saw the impression being formed of them. Unbeknownst to the participant, the “other participant” was always a confederate and thus, the subject was always scanned (and thus the one being evaluated).

### Scan day

On the scan day, all participants arrived at the scanning facility at 12:30 p.m.; they were met by two experimenters and were introduced to the other “participant,” actually a confederate. The participant and confederate interacted for 2 min to establish a rapport, after which point they were placed in separate testing rooms, where they stayed for the first hour. During this time, participants acclimatized to the testing environment and completed socio-demographic and psychological trait questionnaires, including the Beck Depression Inventory—II (Beck et al., 1996), State Trait Anxiety Inventory (STAI) (Spielberger, 1983), Rosenberg Self-Esteem Scale (Rosenberg, 1965), Fear of Evaluation Scale (Leary, 1983), and Mehrabian Sensitivity to Rejection Scale (Mehrabian, 1976). Twenty-five minutes prior to the scan, participants completed an in-house state questionnaire which asked them to provide their impression of the other participant including their feelings of social evaluation (“I feel evaluated by the other participant;” “I feel judged by the other participant”) on a scale ranging from 1 (*not at all*) to 7 (*very much*). They also completed an abridged version of the depressed mood subscale of the Profile of Mood States questionnaire (McNair et al., 1992), which assessed their current feelings of depression, from 0 (*not at all*) to 4 (*extremely*), for the following feelings: unhappy, blue, miserable, sad, discouraged, hopeless, worthless, helpless.

After completing the pre-scan state questionnaires, the participant was reunited with the confederate. At this point, the participant was informed that she was chosen to complete the fMRI scan and have her video interview evaluated by the confederate. While addressing both the participant and the confederate, the experimenter explained the details of the social evaluative task, as well as the full scanning procedure. Specifically, it was explained that the confederate would be outside of the scanner in the control room where she would be watching the participant's video on one screen and providing feedback on how the participant was coming across by using an impressions user interface on another screen (see below for technical details). The participant, on the other hand, was told that she would not be able to see her full video; rather, she would only be shown a couple of clips to remind her of what the confederate was seeing and the rest of the time she would be viewing the impressions user interface with the confederate's feedback. The participant was also asked to report, by pressing buttons on a button box, how she felt in response to receiving the feedback. In addition, the participant was informed that before and after the social evaluative task, she would view evaluations of nature scenes and that she would also undergo a structural imaging scan.

#### Social evaluative task

**Figure 1:**
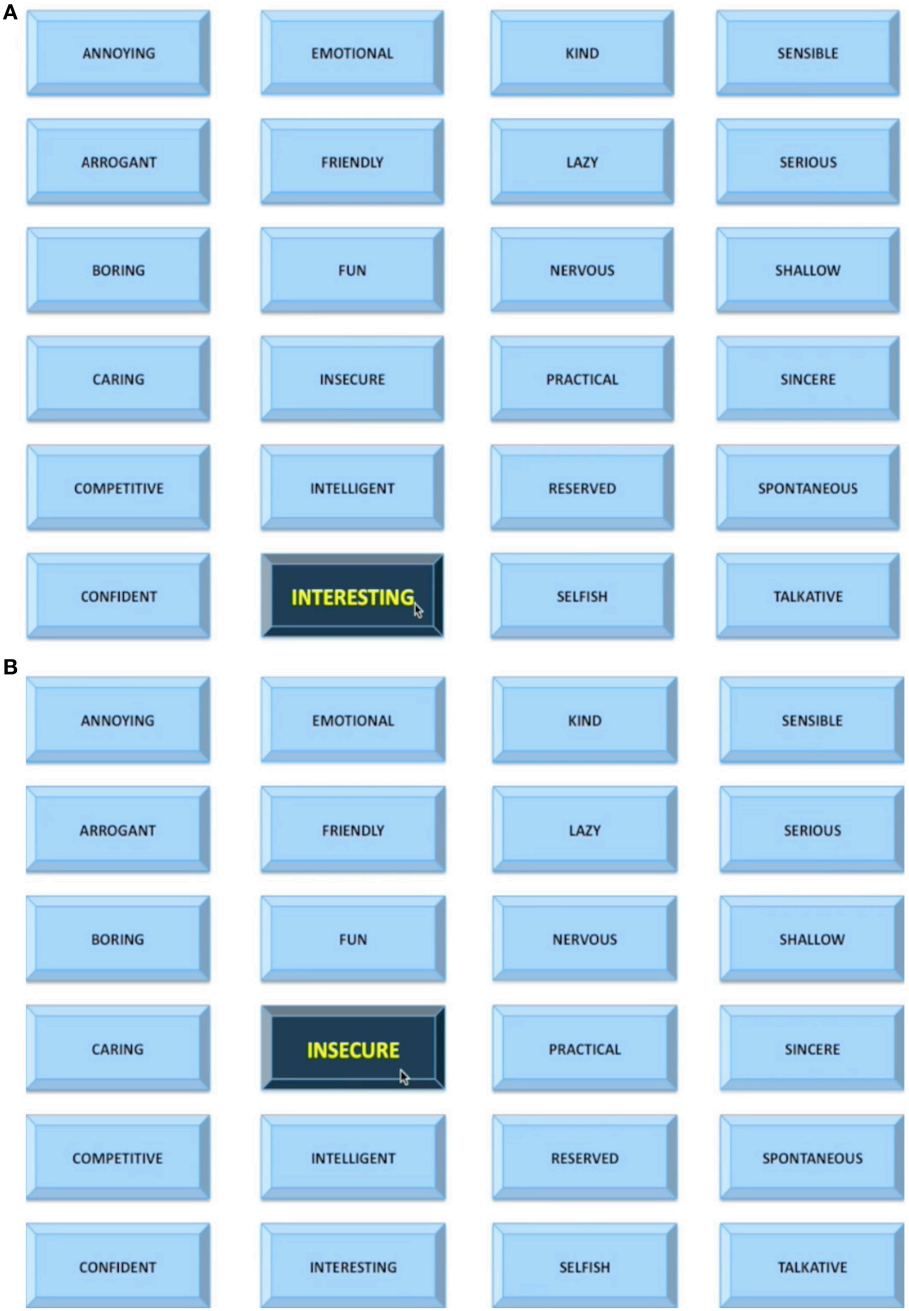
**The “impressions user interface” used in the social evaluative session**. Every 10 s, a participant saw a mouse click over an adjective button indicating the evaluator's rating of the participant's performance in the impressions interview video. The rating could be **(A)** positive, **(B)** negative, or neutral (not shown).

The social evaluative scan task (Eisenberger et al., 2011; Muscatell et al., 2014) started with a short 5 s clip of the start of the participant's own interview, which was then followed by a display of the “impressions user interface”—a 4 × 6 word grid where positive, neutral, and negative adjectives were displayed. Adjectives were selected based on pilot testing with an independent sample of UCLA undergraduates (*N* = 74). The participant saw a mouse moving over the adjectives (believed to be controlled by the evaluator) and, every 10 s, she saw a mouse click over an adjective button indicating the evaluator's rating of the participant's performance in the impressions interview video (Figure 1). Notably, the user interface was in fact a pre-made evaluation video, and all participants saw exactly the same video. During the first part of the evaluation, which lasted 4 min and 28 s, participants saw 6 negative, 9 neutral, and 7 positive adjectives being selected. Then, participants were again shown a short 5 s clip, this time corresponding to the middle of their own interview. This was again followed up by the evaluation video lasting 4 min and 24 s containing 9 negative, 6 neutral, and 8 positive adjectives being selected. Importantly, adjectives in both parts of the evaluation were presented in a pseudorandom order such that no more than two adjectives of the same valence could be presented consecutively. A fixation crosshair (10 s), presented pre- and post-social evaluative task formed the implicit baseline. Participants were instructed that every time they received an evaluation, they were to respond using a button box with four buttons about how they felt at that moment using a 1–4 scale (1 = *really bad*, 4 = *really good*; reverse-coded for manipulation check analyses, so higher numbers indicate feeling worse). Participants were told that the evaluator would have no knowledge of these personal responses.

After the completion of the scanning session, participants returned to the behavioral testing room where they completed the post-scan state questionnaires. Throughout the session, participants also provided biological samples; however, these data are not the subject of the present manuscript.

### Follow-up sessions

At approximately 6 and 12 months following the scanning session, participants who agreed to be contacted for the online follow-up assessments were sent instructions on how to complete several questionnaires, including the BDI-II. Due to subject attrition, the sample sizes for analyses related to the 6-month and 12-month assessments were as follows: in controls, at 6 months, *N* = 26, and at 12 months, *N* = 19; in the previously depressed group, at 6 months, *N* = 13, and at 12 months, *N* = 12.

### Statistical analyses of sociodemographic and psychological trait and state measures

If participants were missing an answer to one item for a given questionnaire, the value for that missing value was replaced either by the mean score of that questionnaire or, if the questionnaire contained subscales, by the mean score of the subscale that the missing item belonged to, for that subject (Osborne, 2013). In the present sample, one HC participant was missing one item on the BDI-II completed on the day of the scan. Furthermore, two participants from the HC group had a missing item each on the Sensitivity to Rejection Scale; one other HC participant had a missing item on the STAI.

For continuous socio-demographic and psychological trait data, an independent *t*-test examined group differences. When data were not normally distributed, group differences were examined using the non-parametric Mann-Whitney U statistic. Group differences on categorical data were assessed using tests of independence (i.e., χ^2^ statistic or Fisher's exact test). A two-way mixed design ANOVA was conducted to examine group differences with respect to changes in psychological state and trait measures over time. Significant interactions were decomposed using simple main effects analyses.

### fMRI image acquisition

Participants were scanned using a Siemens Trio 3.0 Tesla MRI scanner at the UCLA Staglin Center for Cognitive Neuroscience. A T1-weighted MPRAGE anatomical image was acquired with the following specifications: slice thickness = 1 mm, 176 slices, *TR* = 2300 ms, *TE* = 2.98 ms, flip angle = 9°, matrix = 256 × 256, Field-Of-View = 256 mm. In addition, we collected 288 T2-weighted EPI volumes during the social evaluation task with the following specifications: slice thickness = 3 mm, gap = 1 mm, *TR* = 2000 ms, *TE* = 25 ms, flip angle = 90°, matrix = 64 × 64, Field-Of-View = 200 mm.

### fMRI analyses

Neuroimaging data were pre-processed and analyzed using Statistical Parametric Mapping (SPM8; Wellcome Department of Cognitive Neurology, London, UK). In the pre-processing step, images were corrected for head motion, normalized into Montreal Neurologic Institute (MNI) space (resampled at 3 × 3 × 3 mm), and spatially smoothed using an 8 mm full-width-at-half-maximum (FWHM) Gaussian kernel, to increase signal-to-noise ratio.

Next, a general linear model was prepared such that the presentations of each feedback word and the subsequent 11–12 s (until the next word was selected) for each half were modeled as separate blocks and were convolved with a canonical hemodynamic response function. Our regressor-of-interest coded for the type of feedback presented in each half (first-positive, first-neutral, first-negative, second-positive, second-neutral, second-negative), and we included the six motion parameters as covariates. For each model, we applied 128 Hz high-pass filter and autoregressive AR(1) model for serial correlations.

Following the classical model estimation, we computed linear contrasts for each participant that compared BOLD signal during the negative feedback trials to BOLD signal during positive feedback, first for the whole session (i.e., first and second evaluation periods together), and then for the first evaluation period and for the second evaluation period separately. We focused on the contrast of negative—positive words, as this is the most analogous to previous studies investigating social rejection vs. social acceptance (e.g., Eisenberger et al., 2003; Rotge et al., 2015). To examine the main effect of group, contrast images for the whole session were entered into simple *t*-test at the group level for statistical inference. We applied an implicit mask, as well as an explicit whole brain gray matter mask. Note that the main group effect had to be explored within the simple *t*-test framework, as specifying the group contrast within the mixed design flexible factorial framework is not possible. To examine the main effect of evaluation periods, as well as group × evaluation periods interaction contrast images for each period of the evaluation for each participant were entered into flexible factorial analyses at the group level for statistical inference. The following factors were included in the flexible factorial: subject, group (controls vs. previously depressed) and evaluation period (first vs. second bout). Again, we applied an implicit mask, as well as an explicit whole brain gray matter mask. Following the classical model estimation, we examined the contrast for the main effect of time. We also examined the interaction of group by time (0 0 0 0 −1 1 1 −1) reflecting an effect where there would be an increase within the controls and a decrease in the previously depressed, and the inverse of the group by time interaction (0 0 0 0 1 −1 −1 1) representing an effect where there would be a decrease within the controls group and an increase in the previously depressed group.

To evaluate significance of the group main effect, we used a threshold of *p* < 0.005, 104 voxels, which corresponds to a 0.05 false-discovery rate as determined by Monte Carlo simulations conducted in the AFNI program 3dClustSim (parameters: individual voxel *p* = 0.005; 10,000 simulations; FWHM calculated from square root of ResMS at 11.78 × 14.95 × 12.27 mm; mask image file including 43,755 voxels).

To examine the effect of group by evaluation period on processing negative feedback compared to positive feedback, we first conducted a whole-brain analysis and used a threshold of *p* < 0.005, 94 voxels, reflecting 0.05 false-discovery rate as determined by Monte Carlo simulations (3dClustSim parameters: individual voxel *p* = 0.005; 10,000 simulations; FWHM calculated from square root of ResMS at 10.84 × 14.02 × 12.51 mm; mask image file including 43,755 voxels). If this analysis revealed significant effects within the dACC we then used previously defined (Way et al., 2009), independent anatomical regions-of-interest (ROI) based on previous findings (Eisenberger et al., 2011) to investigate dACC association with measures of psychological trait and state characteristics; this was done to ensure that the analyses with respect to change in neural activity and psychological traits and states are not biased (Kriegeskorte et al., 2009).

Specifically, the ROI was constructed in PickAtlas (Maldjian et al., 2003) using templates from the Automated Anatomical Atlas (AAL; Tzourio-Mazoyer et al., 2002). Specifically, the dACC ROI combined Brodman areas 32 and 24, and used a rostral boundary of *y* = +36 based on criteria established by Vogt et al. (2003), and a caudal boundary of *y* = 0 (Way et al., 2009). For the ROI analyses, we first used SPM's *imagecalc* to calculate the change in activity for the negative feedback—positive feedback from the first period to the second period of evaluation (i.e., t2–t1). Then, we extracted parameter estimates from the anatomical ROI using SPM Toolbox MarsBaR. The parameter estimates obtained in this way were then entered into the custom model within the univariate ANCOVA framework to model the group effect, the effect of the covariate (i.e., change in activity in the structural dACC ROI) and the interaction effect between the group factor and the covariate on the dependent variable of interest (e.g., psychological responses to social evaluation, depression symptoms at baseline, 6 and 12 months). A significant interaction effect would reveal that the regression slope between the change in activity in the dACC ROI and the psychological variable of interest differs between the previously depressed and controls. For the psychological state measures, the model was set up to examine the impact of the interaction on the measure taken post the evaluative session while controlling for the pre-scan levels.

## Results

We evaluated BDI-II scores for all participants on the scan day and found that one previously depressed participant scored at clinical levels (BDI-II = 21) and one healthy participant at near-clinical levels (BDI-II = 19). These participants were excluded from subsequent analyses, leaving the total number of subjects per group at 30 for the controls and 16 for the previously depressed group.

### Self-report data

#### Sociodemographic data

The previously depressed participants were older compared to the controls (*M* = 20.1 years vs. *M* = 18.9 years, *U* = 137, *p* = 0.013). Groups did not differ based on their racial or ethnic background (*p*s = 0.67).

#### Psychological traits

At the time of the social evaluative session, previously depressed participants had average BDI-II score within the normal range (*M* = 6.13, *SD* = 5.35); yet, these levels were nevertheless higher compared to controls (*M* = 2.97, *SD* = 3.61, *U* = 148, *p* = 0.031) (Table 1). The previously depressed group also showed higher scores on the Fear of Evaluation scale [*t*_(44)_ = −2.18, *p* = 0.034], higher trait anxiety scores on STAI [*t*_(21.8)_ = −2.9, *p* = 0.008], and lower scores on trait levels of self-esteem [*t*_(44)_ = 2.71, *p* = 0.009]; the groups did not differ with respect to Sensitivity to Rejection [*t*_(44)_ = 1.53, *p* = 0.13] (Table 1).

**Table 1:** **Sociodemographic data and psychological profile of study participants**.

	**Controls *N* = 30 Mean ± Standard Deviation**	**Previously depressed *N* = 16 Mean ± Standard Deviation**
Age	18.9 ± 1.06	20.13 ± 1.78^*^
BDI-II at the evaluative testing session	2.97 ± 3.61	6.13 ± 5.35^*^
BDI-II at 6 months post-testing session	4.23 ± 4.88 (*N* = **26**)	9.00 ± 8.80 (*N* = **13**)
BDI-II at 12 months post-testing session	5.47 ± 5.84 (*N* = **19**)	7.42 ± 6.14 (*N* = **12**)
Mehrabian sensitivity to rejection	−4.80 ± 19.46	−13.06 ± 12.75
Fear of evaluation scale	33.03 ± 7.01	37.62 ± 6.37^*^
State-trait anxiety inventory	33.80 ± 6.53	41.94 ± 10.16^*^
Rosenberg self-esteem	57.6 ± 8.2	49.9 ± 10.68^*^
**RACIAL/ETHNIC BACKGROUND**		
White	6	5
Asian/Filipino/Polynesian	7	4
Latino	7	2
Middle Eastern/East Indian	2	0
Other/mixed	8	5

BDI-II, Beck Depression Inventory-II;

* *p < 0.05 compared to controls. Note the reduction in sample size, written in bold, for analyses involving BDI-II at 6 and 12 months*.

A Group (previously depressed, controls) by Time (baseline, 6, 12 months) ANOVA, revealed a significant Group effect [*F*_(1, 29)_ = 6.17, *p* = 0.019, Partial η^2^ = 0.18], such that the previously depressed participants had overall higher BDI-II scores compared to control participants (Table 1). No other effects (Time, or Time by Group interaction) were significant (*p*s > 0.25).

#### Moment-to-moment responses to the social evaluative task

To ensure that the evaluative task was having the intended effect, we examined whether participants' feelings in response to the evaluative feedback varied depending upon whether they were seeing negative, positive, or neutral adjectives in the scanner. A three-way mixed ANOVA [Group (previously depressed vs. controls) by Feedback Type (positive, negative, neutral) by Evaluation period (first bout vs. second bout of evaluation)] yielded a significant main effect of Evaluation period, revealing that all participants felt worse over time [*F*_(1, 44)_ = 12.21, *p* = 0.001, Partial η^2^ = 0.22]. We also observed a main effect of Feedback Type [*F*_(1.39, 61.14)_ = 150.34, *p* < 0.001, Partial η^2^ = 0.77], confirming that all participants felt worse in response to negative compared to neutral feedback [*t*_(45)_ = 14.97, *p* < 0.001] and worse in response to neutral compared to positive feedback [*t*_(45)_ = 8.21, *p* < 0.001]. Finally, we observed a significant Feedback Type by Group interaction [*F*_(1.39, 61.14)_ = 6.05, *p* = 0.009, Partial η^2^ = 0.12]. Decomposing the interaction revealed that in response to positive feedback, the previously depressed participants endorsed greater levels of negative feelings (*M* = 1.90; *SD* = 0.65) compared to the controls (*M* = 1.40; *SD* = 0.42) [*F*_(1, 44)_ = 11.45, *p* = 0.002], but there were no differences for negative [*F*_(1, 44)_ = 0.13, *p* = 0.72] or neutral feedback [*F*_(1, 44)_ = 0.55, *p* = 0.46]. No other effects (i.e., main effect of Group, Group × Evaluation period, Evaluation period × Feedback Type, Group × Feedback × Evaluation period interaction) were significant.

#### Changes in psychological states from pre- to post-evaluation

##### Feelings of social evaluation

**Figure 2:**
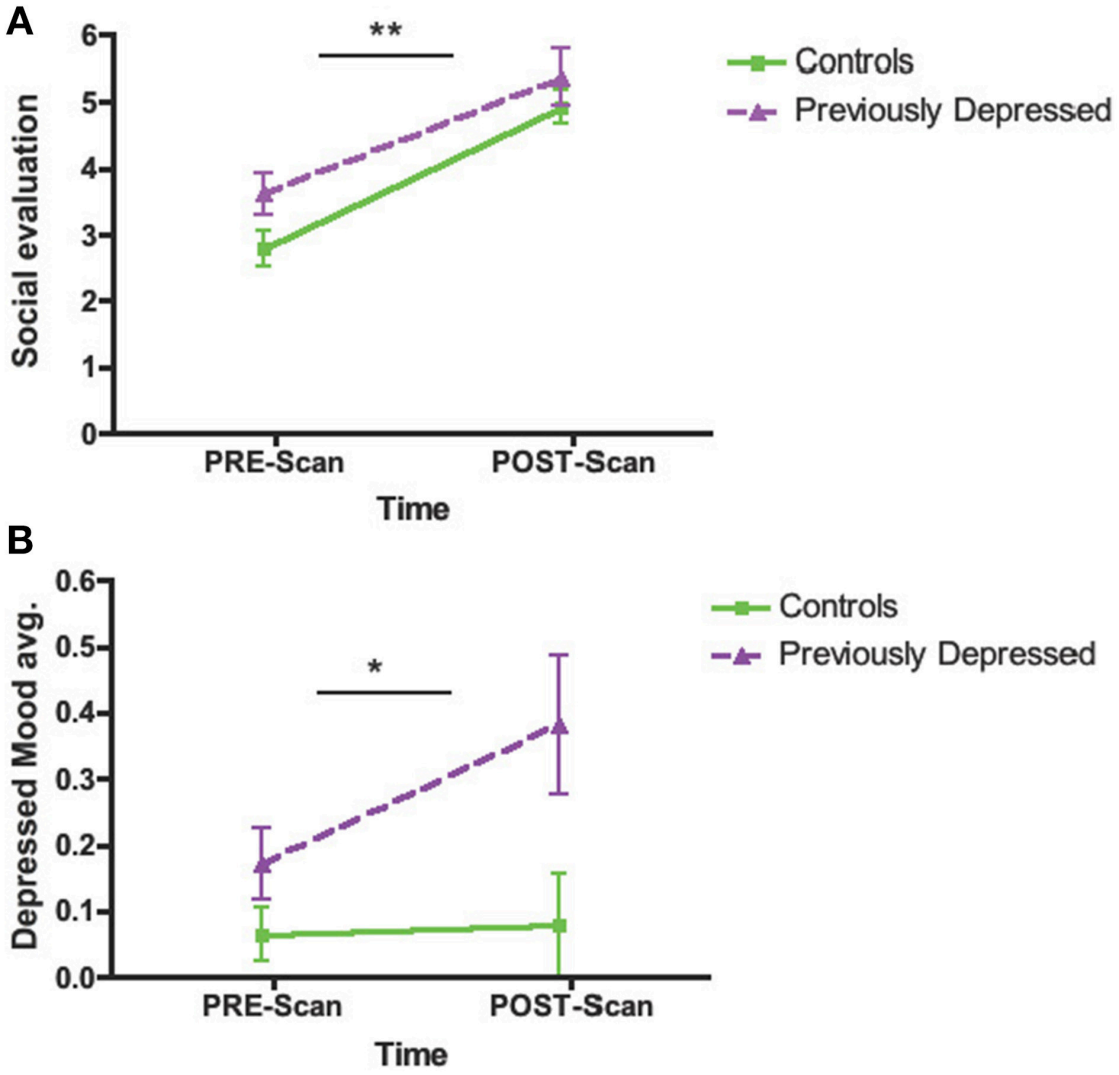
**Change in Psychological States from Pre- to Post-scan during the Social Evaluative Session. (A)** Feelings of social evaluation. **(B)** Depressed mood. Main effect of Time shown. ^**^p < 0.001; ^*^p < 0.05.

To examine whether the task was successful in increasing feelings of social evaluation, we conducted a two-factor mixed-design ANOVA (Group by Time pre- vs. post-scan). The analysis showed only a significant effect of Time reflecting increased feelings of social evaluation post-task (*M* = 5.09; *SD* = 1.50) compared to pre-task (*M* = 3.09; *SD* = 1.44) in all participants [*F*_(1, 44)_ = 84.47, *p* < 0.001, Partial η^2^ = 0.66; Figure 2A]. No other effects (Group, Group × Time interaction) were significant (*p*s > 0.12).

##### Depressed mood

A Group by Time (pre- vs. post-scan) ANOVA for depressed mood revealed a significant main effect of Group [*F*_(1, 44)_ = 5.87, *p* = 0.02, Partial η^2^ = 0.12], showing overall greater levels of depressed mood in previously depressed participants compared to controls. We also observed a main effect of Time [*F*_(1, 44)_ = 4.17, *p* = 0.047, Partial η^2^ = 0.09] reflecting an increase in depressed mood over time. Importantly, the Time effect was primarily driven by a bigger increase in depressed mood over time in the previously depressed group as suggested by the tendency for Group by Time interaction [*F*_(1, 44)_ = 3.29, *p* = 0.077, Partial η^2^ = 0.07; Figure 2B].

### Neuroimaging data

#### Whole brain analysis

**Figure 3:**
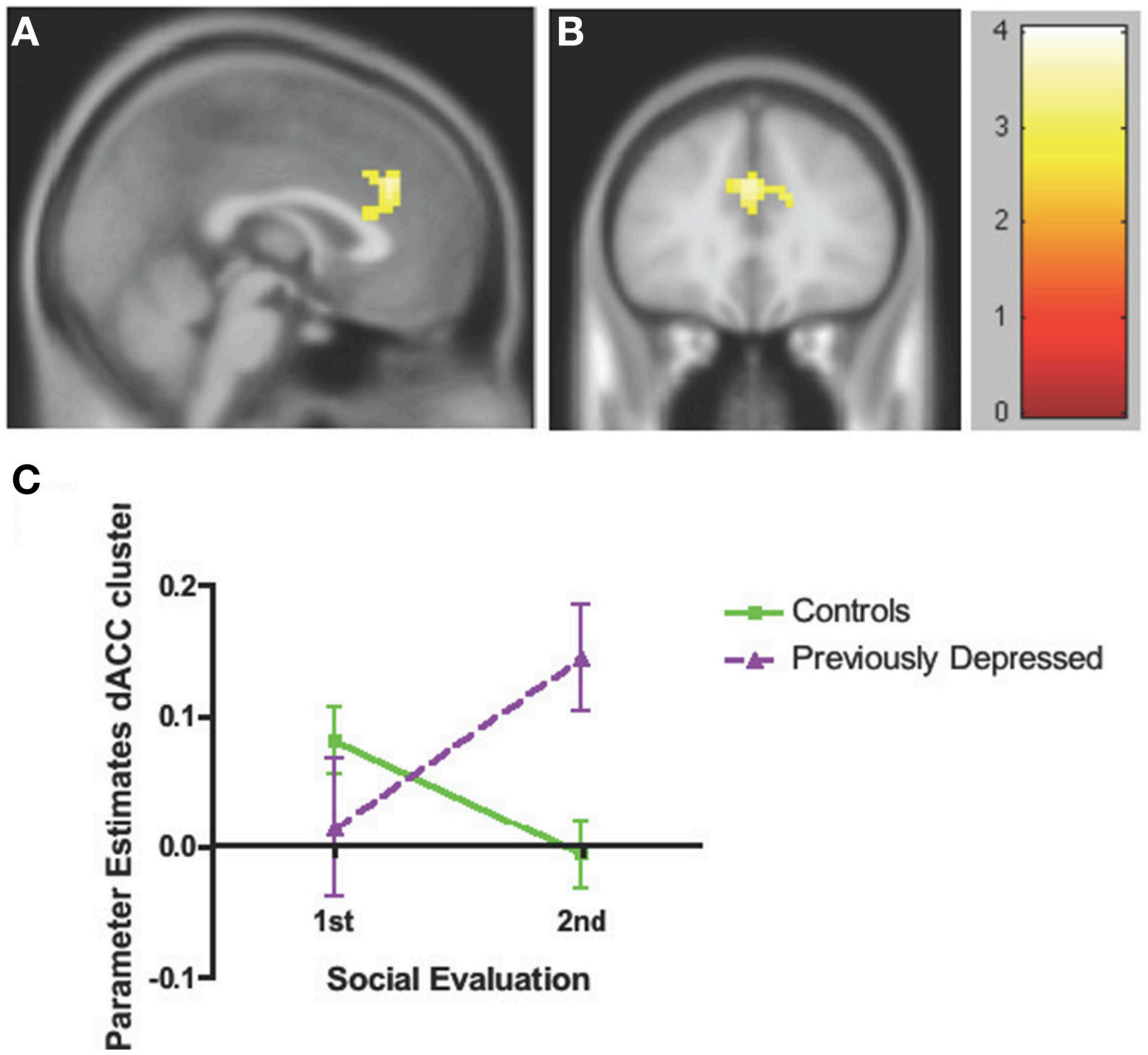
**Whole brain analyses of group × time interaction reflecting effect where neural activity increases over time in the previously depressed women but decreases in controls. (A)** Sagittal view revealing a significant cluster in dorsal anterior cingulate cortex (dACC). **(B)** Coronal view of the same dACC cluster. **(C)** Parameter estimates extracted from the dACC cluster showing that within the control group activity within the dACC decreases, while in the previously depressed group, it increases. Note that extracted parameter estimates from this dACC cluster are taken from non-independent voxels and thus are used here simply to illustrate the observed effect at the whole brain level.

The whole brain Group effect revealed no significant activations in the dACC or in any other neural regions. Furthermore, there was no significant effect of the Evaluation period (first vs. second bout of evaluation). However, there was a significant Group by Evaluation period interaction effect only in the dACC (cluster = 127, corrected *p* < 0.05, MNI coordinates *x* = 12, *y* = 20, *z* = 31; Figures 3A,B; Table 2), such that there was a decrease over the two bouts of evaluation in dACC activity within the control group and an increase in the previously depressed group. Lowering the voxel threshold to *p* < 0.005, and the cluster extent to 40, revealed additional regions including anterior insula (MNI coordinates, *x* = −27, *y* = 17, *z* = 1; see Table S1).

**Table 2:** **Whole brain analyses of group × time interaction reflecting effect where neural activity increases over time in the previously depressed women but decreases in controls**.

	**Anatomical region**	**Brodmann Area**	***x***	***y***	***z***	***t***	***k***
**GROUP × TIME**							
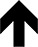 **Previously Depressed**							
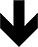 **Controls**							
***Whole Brain***							
	dACC	24	12	20	31	4.04	127
		*32*	−*12*	*26*	*28*	*3.86*	
		*32*	*0*	*32*	*31*	*3.63*	

*Effects are significant at p < 0.05 corrected. Montreal Neurological Institute coordinates; coordinate in italics connote peaks within a same cluster; dACC, dorsal anterior cingulate cortex*.

In order to decompose the whole brain interaction effect for dACC, we extracted parameter estimates directly from the significant dACC cluster and entered these into SPSS. These analyses revealed that the activity within dACC was reduced over the two evaluation bouts among the controls [*F*_(1, 44)_ = 6.49, *p* = 0.014] but increased among the previously depressed participants [*F*_(1, 44)_ = 7.65, *p* = 0.008]. In addition, the groups did not differ with respect to activity in dACC during the first bout of the evaluation [*F*_(1, 44)_ = 1.65, *p* = 0.21], but previously depressed (vs. healthy controls) had higher levels of activity during the second bout of the evaluation [*F*_(1, 44)_ = 10.30, *p* = 0.002] (Figure 3C). It is important to note that extracted parameter estimates from this dACC cluster are taken from non-independent voxels, which can lead to biases in additional statistical analyses. Thus, the extracted parameter estimates and these additional analyses are used simply to illustrate which effect in which group is driving the observed effect at the whole brain level.

To examine group differences with respect to how change in dACC activity was associated with changes in psychological responses to the task as well as depressive symptoms at baseline, 6 and 12 months, we extracted parameter estimates from an independent anatomical dACC ROI and conducted the statistical analyses using SPSS (see Methods and Materials for details). We used the independent anatomical dACC ROI in order to ensure that these correlational analyses were independent of the whole brain group × evaluation period interaction analyses (Kriegeskorte et al., 2009).

#### Correlations between changes in dACC ROI activity over evaluation periods and self-reported psychological responses to the task

There were no group differences in how changes in dACC activity correlated with changes in either feelings of social evaluation or depressed mood.

#### Correlations between changes in dACC ROI activity over evaluation periods and depressive symptoms at baseline, 6 months, and 12 months post-task

##### Baseline depressive symptoms

**Figure 4:**
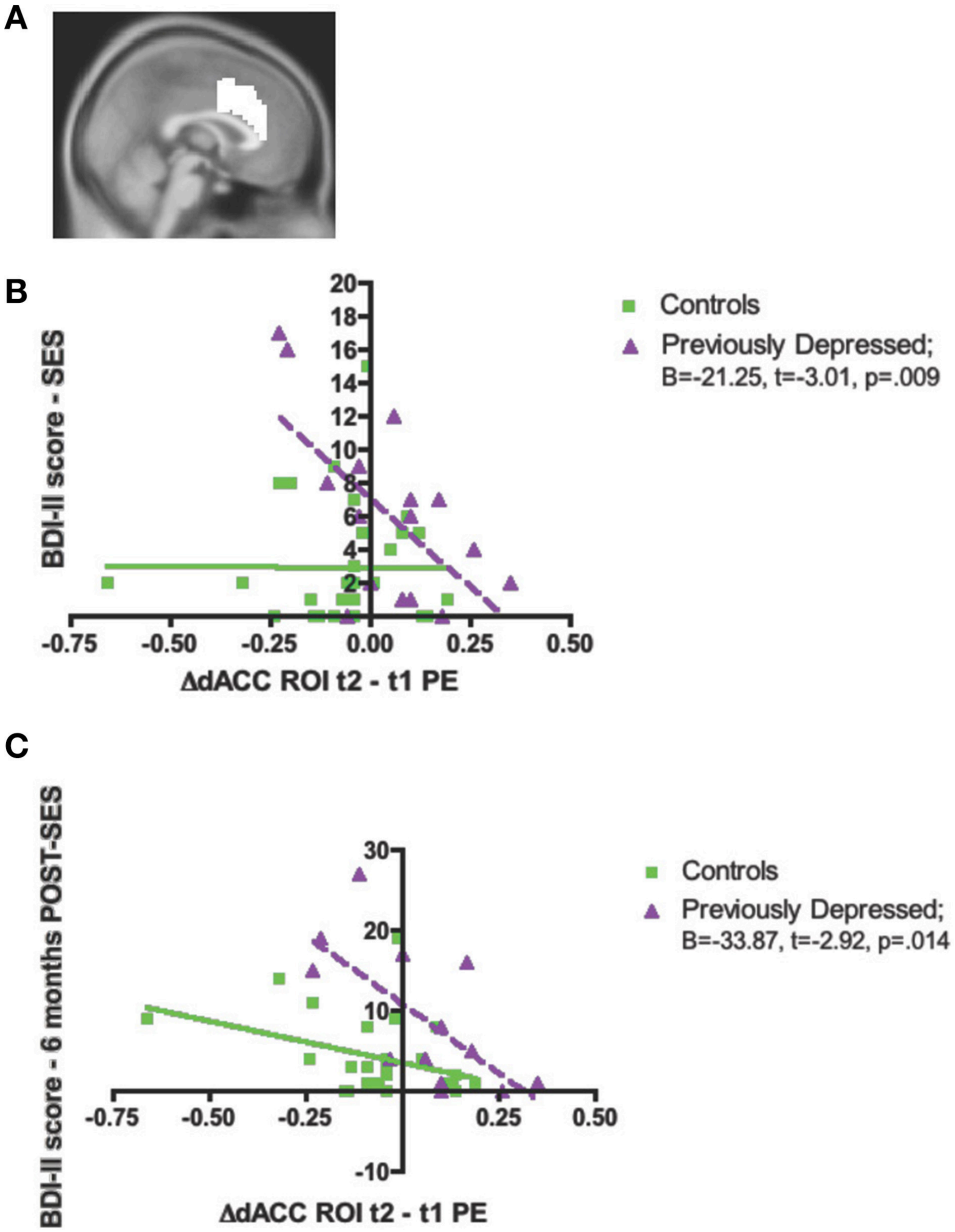
**Significant associations between the increase in activity over time in dorsal anterior cingulate cortex (dACC) independent, structural region of interest (ROI), and psychological measures in the previously depressed women compared to controls. (A)** Sagittal view of the independent, structural dACC region of interest used to extract parameter estimates for subsequent analyses; **(B)** Beck Depression Inventory-II (BDI-II) score at the social evaluative session (SES); **(C)** BDI-II score at 6 months following the social evaluative session (SES).

We observed a significant interaction effect between Group and Change in activity in the dACC ROI (Figure 4A) on baseline depressive symptoms (evaluated via the BDI-II), [*F*_(1, 42)_ = 7.56, *p* = 0.009, Partial η^2^ = 0.15]. Thus, among the previously depressed, the greater the increase in activity in dACC over the two bouts of the evaluation, the *lower* the depressive symptoms on the day of the evaluation (*B* = −21.25, *t* = −3.01, *p* = 0.009), but no relationship was found among the controls (*B* = −0.015, *t* = −0.004, *p* = 0.99; Figure 4B).

##### Depressive symptoms at 6 and 12 months post-task

Finally, we also investigated whether there was a Group difference with respect to regression slopes between the Change in the dACC ROI and the BDI-II scores assessed at 6 and 12 months. We found a significant interaction effect for 6 months [*F*_(1, 35)_ = 4.56, *p* = 0.04, Partial η^2^ = 0.12 Figure 4C], but not 12 months [*F*_(1, 27)_ = 1.98, *p* = 0.17, Partial η^2^ = 0.07]. The significant interaction for the BDI-II levels at 6 months revealed that, only among the previously depressed, the greater increase in activity in the dACC over the two bouts of evaluation, the *lower* the BDI-II score at 6 months post-social evaluative session (previously depressed group: *B* = −33.87, *t* = −2.92, *p* = 0.014; controls group: *B* = −10.23, *t* = −1.93, *p* = 0.07; Figure 3C).

To examine whether the relationship between changes in dACC activity and depressive symptoms at 6 months in the previously depressed participants was independent of participants' depressive symptoms at the time of the social evaluative session, we conducted a hierarchical regression. We first entered the BDI-II scores at the time of the social evaluative session and, in the next step, entered the parameter estimates from the dACC ROI. There was a significant change in the fitness of the model upon the introduction of the dACC ROI (*F* change = 5.02, *p* = 0.049), with only the second model that included both BDI-II scores and dACC ROI being significant [*F*_(2, 10)_ = 4.14, *p* = 0.049]. Specifically, the second model revealed that change in activity in dACC ROI significantly contributed to the BDI-II at 6 months, *t* = −2.24, *p* = 0.049, controlling for baseline BDI-II levels (Table 3).

**Table 3:** **Hierarchical regression reveals significant and unique contribution of increase in activity in dorsal anterior cingulate cortex (dACC) to depression levels at 6 months following the social evaluative session in the previously depressed women**.

	***b***	**SE *B***	**β**	***p***
**MODEL 1**				
Constant	4.22	3.861		0.298
BDI-II at testing session	0.683	0.442	0.422	0.151
**MODEL 2**				
Constant	13.659	5.352		0.029
BDI-II at testing session	–0.324	0.587	–0.201	0.593
ΔdACC t2-t1	–41.74	18.624	–0.814	0.049

*Adjusted R^2^ for Model 2 = 0.344; F change between Model 2 vs. Model 1, p = 0.049; Δ, change in activity in dACC from first bout (t1) to second bout (t2) of social evaluation; BDI-II, Beck Depression Inventory-II*.

## Discussion

The present study aimed to investigate neural mechanisms underlying the association between repeated exposure to social evaluation and depressive symptoms in psychiatrically healthy and previously depressed young women. Although, both groups reported increases in feelings of social evaluation in response to a brief socially evaluative stressor, we found evidence that previously depressed participants experienced this social evaluation in a unique way. Namely, the previously depressed (compared to controls) tended to show increased levels of depressed mood in response to the social evaluation. In addition, previously depressed compared to the controls showed more negative feelings in response to positive feedback, which is consistent with the role of anhedonia in depression (e.g., Eshel and Roiser, 2010; Beevers et al., 2013). Overall, these findings are in line with the idea that the previously depressed represent a population vulnerable to developing depression.

With regard to neural activity, we did not observe an overall group effect or time effect. However, as expected, the previously depressed participants did show increased activity over repeated bouts of social evaluation within the dACC in response to negative compared to positive feedback, while controls showed a decline in this contrast. Interestingly, and surprisingly, this increase within the dACC in the previously depressed was linked with lower levels of depressive symptoms at baseline, and most notably, with lower levels of depressive symptoms 6 months after the social evaluative session—an effect that held even after controlling for baseline depression levels. Thus, those previously depressed women who showed increases over time in dACC responses to negative compared to positive feedback seemed to show traces of resilience with respect to their current psychological state and well as their depression symptoms 6 months later.

It has been suggested that the dACC plays an important role in responding to social exclusion (Kawamoto et al., 2012), with experiences of negative social evaluation or rejection consistently being associated with overall heightened activity in the dACC in healthy samples (Eisenberger et al., 2003, 2011; Kross et al., 2011; Rotge et al., 2015). Further, in healthy samples, this engagement of dACC was found to wane over the course of playing a computerized game of social exclusion (Crowley et al., 2009; Moor et al., 2012; Kawamoto et al., 2013; Themanson et al., 2013). Therefore, the decrease over time in dACC activity in response to negative compared to positive feedback in the healthy sample observed here could represent an adaptive response to negative social evaluation. Indeed, this pattern of response is consistent with the idea that if one mounts a physiological response to a personally-relevant stressful situation, that response should be followed by a successful recovery, if it is to be adaptive (Fredrickson et al., 2003; Tugade and Fredrickson, 2004).

The previously depressed group did not show difference in the overall activity in dACC, which suggests that abnormalities in overall dACC activity previously observed in MDD patients (Hamilton et al., 2011; Crocker et al., 2013; Graham et al., 2013; Ubl et al., 2015) could be a characteristic of being in a depressive state (Hamilton et al., 2011). The increase in dACC activity over the course of the social evaluation task, however, is similar to what was previously observed in MDD patients (Waugh et al., 2012). Still, it is not yet clear what function the dACC is serving during the task. Waugh and colleagues proposed that the resurgence of medial frontal cortex activity including dACC over the course of their stress paradigm in MDD patients may represent either rumination about negative aspects of the stress task or, alternatively, generation of arousal necessary for anticipated effort in performing the task (Waugh et al., 2012). Future work will be needed to more fully examine these alternatives.

Surprisingly, in the current study, increased dACC activity over the two social evaluation periods was associated with *lower* depressive symptoms at baseline as well as at 6 months following the social evaluative session. Although, running contrary to what one would expect from the previous findings of heightened dACC activity during a given experience of social evaluation, these findings are in line with previous studies showing that heightened risk for developing depression could be linked with a blunted neural response to emotional contexts (McCabe et al., 2012; Kujawa et al., 2014; Chester et al., 2015), a phenomenon that has also been observed in persons experiencing a depressive episode (e.g., Miller et al., 2015; Ubl et al., 2015). Specifically, a blunted neural response in the dACC to both negative and positive stimuli (e.g., food or monetary) has been observed in children and young adults at heightened vulnerability to develop depression due to family history (McCabe et al., 2012; Kujawa et al., 2014). In addition, another study in young adults with heightened subclinical levels of alexythimia, a condition with strong links to depression (Honkalampi et al., 2000), revealed that participants who tended to have difficulty identifying their feelings felt more rejected on a daily basis in part because of diminished dACC activity during social rejection experiences (Chester et al., 2015). It was proposed that the blunted dACC response resulted in a failure of these individuals to adjust their behavioral tendencies to achieve social inclusion in the future (Chester et al., 2015). Therefore, it is possible that increased dACC activity over time may represent a form of emotional context sensitivity, which is considered an adaptive emotional response even among those remitted from depression (Rottenberg et al., 2005; Waugh and Koster, 2014), and therefore may constitute a sign of resilience. As we did not include a third bout of evaluation, we could not evaluate whether these individuals were also able to show the appropriate adaptive recovery of this increased response; this should be evaluated in future studies. Future work is needed to better understand why increasing neural sensitivity over time in previously depressed participants was related to better subsequent outcomes.

Although, the current study reveals important details with respect to the role of the dACC during the processing of *repeated* social evaluation and vulnerability/resilience to depression, it does have some notable limitations. First, the study has uneven sample sizes per study groups with small sample size affecting the previously depressed group; this is due to difficulty in recruiting previously depressed participants who at the time of testing had a healthy psychological profile. Nevertheless, the sample size within the previously depressed group is adequate and within the guidelines for the employed statistical tests (Field, 2005). Second, due to careful selection of the previously depressed sample in order to limit sources of variability and the fact that we investigated a university student sample, it is possible that the current results are not generalizable to all participants with a past history of depression. Study results should be replicated in a larger, community-based sample. Furthermore, the social evaluative task had only two repetitions of social evaluations, which provides only limited insight into the nature of temporal dynamics of dACC activity; future studies should include multiple repetitions of social evaluative sessions.

Overall, the study revealed that while young women with a past history of MDD tend to be particularly sensitive to repeated bouts of social evaluation in terms of its effects on depressed mood, those who showed increases in dACC activity over the course of the social evaluative session to negative compared to positive feedback showed lower depressive symptoms at baseline and 6 months later. Changes in dACC activity over repeated bouts of social evaluation may be an important mechanism underlying the association between experiences of repeated social evaluation and individual differences in continued resilience and vulnerability to depression. Given that the change in dACC activity *over time* in response to negative evaluation reveals a new dimension to the role of the dACC in processing exclusion and its association with mental health outcomes in a sample with distinct vulnerability to depression, further investigation of the dynamics of dACC response to negative social evaluation is warranted.

## Author contributions

KD: study design, study execution, analyses, manuscript writing and editing. GS: study design, study execution, manuscript editing. KM: study design, study execution, manuscript editing. MI: study design, manuscript editing. NE: study design, manuscript writing and editing.

## Funding

This work was supported by a National Alliance for Research on Schizophrenia and Depression (NARSAD) Young Investigator Award (NE), a UCLA Cousins Center for Psychoneuroimmunology Seed Grant (NE), a UCLA Clinical & Translational Science Institute (CTSI) Seed Grant (NE), the NIH/National Center for Advancing Translational Science (NCATS) UCLA CTSI Grant Number UL1TR000124, the UCLA Older Americans Independence Center Inflammatory Biology Core (funded by NIA/NIH Grant Number AG028748), the National Science Foundation Graduate Research Fellowship Program (KM), NIH Pre-Doctoral Institutional Training Grant T32 MH015750 (KM), and the Canadian Institutes of Health Research Postdoctoral Fellowship program (KD).

### Conflict of interest statement

The authors declare that the research was conducted in the absence of any commercial or financial relationships that could be construed as a potential conflict of interest.
