# Supplementary material for: Dorsal Anterior Cingulate Cortex Responses to Repeated Social Evaluative Feedback in Young Women with and without a History of Depression
Source: Front Behav Neurosci. 2016 Mar 31;10:64. doi: 10.3389/fnbeh.2016.00064 (PMC4815251; doi:10.3389/fnbeh.2016.00064)
Supplement: Supplementary file 1 [file Table1.DOCX]

**Table S1:** **Whole brain analyses of group x time interaction thresholded at p<.005, 20 voxels reflecting effect where neural activity increases over time in the previously depressed women but decreases in controls**

|  | **Anatomical region** | ***x*** | ***y*** | ***z*** | ***t*** | ***k*** |
| --- | --- | --- | --- | --- | --- | --- |
| **GROUP x TIME** |  |  |  |  |  |  |
| **🡹Previously Depressed**  **🡻Controls** |  |  |  |  |  |  |
| *Whole Brain* |  |  |  |  |  |  |
|  | dACC | 12 | 20 | 31 | 4.04 | 127 |
|  |  | *-12* | *26* | *28* | *3.85* |  |
|  |  | *0* | *32* | *31* | *3.63* |  |
|  |  |  |  |  |  |  |
|  | Right cuneus | 6 | -61 | 10 | 3.68 | 85 |
|  |  | *18* | *-73* | *13* | *3.63* |  |
|  |  | *21* | *-82* | *7* | *3.37* |  |
|  |  |  |  |  |  |  |
|  | Left anterior insula | -27 | 17 | 1 | 3.68 | 40 |

Montreal Neurological Institute coordinates; coordinate in italics connote peaks within a same cluster; dACC: dorsal anterior cingulate cortex
